# Kupffer cells of cirrhotic rat livers sensitize colon cancer cells to Fas-mediated apoptosis

**DOI:** 10.1054/bjoc.2000.1737

**Published:** 2001-05

**Authors:** E Song, J Chen, N Ouyang, M Wang, M S Exton, U Heemann

**Affiliations:** Departments of Nephrology and Medical Psychology, University Hospital Essen, Hufelandstr. 55, 45122 Essen, Germany; Department of Surgery, Sun-Yat-Sen Memorial Hospital, Sun-Yat-Sen University of Medical Science, 510120 Guangzhou, Peoples Republic of China

**Keywords:** colon neoplasms, metastasis, liver cirrhosis, Kupffer cells, apoptosis

## Abstract

Metastasis of colorectal carcinomas rarely occurs in cirrhotic livers. Our study investigated the influence of activated Kupffer cells from cirrhotic rat livers on hepatic colonization and FasR-mediated apoptosis of colon cancer cells. A rat colon cancer cell line, RCN-9, was used to inoculate rat livers. Treatment with conditioned media of Kupffer cells isolated from CCl _4_-induced cirrhotic rat livers (cirrhotic KCM) significantly reduced the incidence of hepatic colonization of RCN-9 cells. In vitro cytotoxicity of Kupffer cells and tumour infiltrating lymphocytes (TILs) on RCN-9 cells was evaluated using [^3^H]-release assay. RCN-9 cells were resistant to cytotoxicity mediated by cirrhotic Kupffer cells, but were sensitized to TIL-mediated killing after treatment with cirrhotic KCM. The specific killing induced by TILs was FasR-mediated, as it was inhibited by ZB4, an antagonistic anti-FasR antibody. In agreement, cirrhotic KCM increased recombinant Fas ligand-induced apoptosis of RCN-9 cells, and up-regulated FasR expression on RCN-9 cells as evaluated by RT-PCR and flow cytometry. These findings suggest that Kupffer cells in cirrhotic livers sensitize metastatic colon cancer cells to FasR-mediated apoptosis by up-regulating the receptors, which thus prepare them to be eliminated by infiltrating lymphocytes. © 2001 Cancer Research Campaign http://www.bjcancer.com


*British Journal of Cancer* (2001) **84**(9), 1265–1271
© 2001 Cancer Research Campaign
doi: 10.1054/ bjoc.2001.1737, available online at http://www.idealibrary.com on IDEAL®

http://www.bjcancer.com

### Kupffer cells of cirrhotic rat livers sensitize colon cancer cells to Fas-mediated apoptosis

**E Song[1,3], J Chen[3], N Ouyang[1], M Wang[1,3], MS Exton[1,2] and U Heemann[1]**

Departments of [1]Nephrology and [2]Medical Psychology, University Hospital Essen, Hufelandstr. 55, 45122 Essen, Germany; and [3]Department of Surgery, Sun-Yat-Sen Memorial Hospital, Sun-Yat-Sen University of Medical Science, 510120 Guangzhou, Peoples Republic of China

**Summary** Metastasis of colorectal carcinomas rarely occurs in cirrhotic livers. Our study investigated the influence of activated Kupffer cells from cirrhotic rat livers on hepatic colonization and FasR-mediated apoptosis of colon cancer cells. A rat colon cancer cell line, RCN-9, was used to inoculate rat livers. Treatment with conditioned media of Kupffer cells isolated from $CCl_4$-induced cirrhotic rat livers (cirrhotic KCM) significantly reduced the incidence of hepatic colonization of RCN-9 cells. In vitro cytotoxicity of Kupffer cells and tumour infiltrating lymphocytes (TILs) on RCN-9 cells was evaluated using [$^3$H]-release assay. RCN-9 cells were resistant to cytotoxicity mediated by cirrhotic Kupffer cells, but were sensitized to TIL-mediated killing after treatment with cirrhotic KCM. The specific killing induced by TILs was FasR-mediated, as it was inhibited by ZB4, an antagonistic anti-FasR antibody. In agreement, cirrhotic KCM increased recombinant Fas ligand-induced apoptosis of RCN-9 cells, and up-regulated FasR expression on RCN-9 cells as evaluated by RT-PCR and flow cytometry. These findings suggest that Kupffer cells in cirrhotic livers sensitize metastatic colon cancer cells to FasR-mediated apoptosis by up-regulating the receptors, which thus prepare them to be eliminated by infiltrating lymphocytes. © 2001 Cancer Research Campaign http://www.bjcancer.com

**Keywords**: colon neoplasms; metastasis; liver cirrhosis; Kupffer cells; apoptosis

Metastases of colorectal cancers into injured livers, such as chronic viral hepatitis (Utsunimoiya et al, 1999), liver cirrhosis (Seymour and Charnley, 1999) and fatty liver (Hayashi et al, 1997), are rare, with an incidence ranging from 0–8%. Recent studies suggest that in these hepatic disorders, changes in liver-associated immunity may play an important role in hindering hepatic metastasis formation. However the mechanism remains illusive (Okuno et al, 1998).

For metastatic tumour cells to become established and develop into overt lesions, they must first evade immune surveillance at the secondary sites. Cytotoxic T lymphocytes (CTLs) and natural killer (NK) cells constitute the first line of cellular defence against invading cancer cells in the liver. Activated CTLs and NK cells may conduct cytotoxicity on target cells by Fas receptor (FasR) and Fas ligand (FasL) interaction (Nagata, 1997). Although lymphocytes infiltrating the metastatic colorectal cancers in livers express high levels of FasL, the metastatic cancer cells can evade FasR-mediated cell killing induced by CTLs. It has been demonstrated that highly metastatic colorectal cancer cells lack FasR expression, and thus are resistant to FasL-induced apoptosis. This was suggested to be a pre-requisite for the colonization of the malignant cells in the liver (Yoong et al, 1999).

However, expression of Fas receptors on colorectal cancer cells can be up-regulated by pro-inflammatory cytokines produced by macrophages upon activation, which thus enhance the sensitivity of tumour cells to CTL-mediated cytotoxicity (Möller et al, 1994; Krammer et al, 1998). This phenomenon underlines the possible concerting role of macrophages and CTLs in controlling hepatic metastasis of colorectal cancers. In a rat model of experimentally induced hepatic metastases, a prominent increase in the number of Kupffer cells, macrophages residing in liver, was observed, suggesting that these cells were involved in the immune response against tumour cell deposits in the liver (Thomas et al, 1993). Nevertheless, despite an increase in Kupffer cells, together with FasL-positive CTLs, these cells failed to contain the development of metastases. This is because the Kupffer cells residing in normal livers were not activated, as evaluated by various activation criteria. Hence, it was suggested that Kupffer cells in normal livers were not effective in the defence against metastasis of colorectal cancers (Griffini et al, 1996).

In cirrhotic livers, however, Kupffer cells are activated and release constitutive amount of proinflammatory factors (Gressner, 1996). The activated Kupffer cells assorting with FasL expressing CTLs may be able to eliminate metastatic tumour cells in cirrhotic livers. But the interaction between colon cancer cells and pre-activated Kupffer cells from cirrhotic livers has not yet been studied.

Therefore, the aim of the present study was to explore the influence of activated Kupffer cells from cirrhotic rat livers on hepatic colonization of colon cancer cells, and to investigate the regulatory effect of these macrophages on FasR expression as well as FasR-mediated apoptosis in colon cancer cells.

### MATERIALS AND METHODS

### Animals and liver cirrhosis model

Male Fischer rats (220–250 g; Charles River, Sulzfeld, Germany) were used throughout the experiments. All animals were kept under routine laboratory conditions and received care in compliance with the institution's guidelines. Liver cirrhosis was induced by $CCl_4$ in the presence of phenobarbital as previously described (Armendáriz-Borunda et al, 1989). Cirrhosis of the liver was confirmed by histological examination. Untreated animals were used as healthy controls.

*Received 18 August 2000*
*Revised 25 January 2001*
*Accepted 30 January 2001*

*Correspondence to:* U Heemann

### Preparation of Kupffer cells

Kupffer cells were isolated from $CCl_4$-induced liver cirrhosis and from healthy control rats as described elsewhere (Armendáriz-Borunda et al, 1989). Briefly, after perfused with Gey's balanced saline solution (GBSS) solution supplemented with 0.2% pronase E and 0.8 μg $ml^{-1}$ DNase (Sigma Chemical Co, St Louis, MO, USA) via the portal vein, the liver was removed and cut into small pieces, which were then incubated at 37˚C for 45 min with the same solution. Hepatocytes were then removed by centrifugation. The supernatant was saved and the isolation of Kupffer cells was achieved by centrifugation at 600 × ***g***, 4˚C, for 15 min in a 17.5% solution of metrizamide (Accurate Chemicals and Scientific Co, Westbury, NY) in GBSS. The interface layer containing Kupffer cells was isolated and washed in PBS. Further purification was achieved by allowing the cells to adhere to plastic culture plates at 37˚C for 2 h. Nonadherent cells were removed by washing, and the adhered Kupffer cells were cultured in Dulbecco's modified Eagle medium (DMEM) containing 10% fetal calf serum (FCS) at 37˚C for 24 h for further studies. The purity of Kupffer cells after 24 h culture as assayed by α-naphthyl acetate esterase (Sigma) staining was greater than 95%. The viability of cells was always greater than 95% as determined by trypan blue exclusion. Kupffer cell-conditioned media (KCM) was prepared by removing medium supernatant from Kupffer cells after 24 h of culture, which was then filtered through Millipore (0.22 μm) for further study.

### Colon cancer cell line and treatment

The colon adenocarcinoma cell line RCN-9, a dimethylhydrazine-induced colon carcinoma of Fischer rats (Inoue et al, 1991), was obtained from ATCC (Rockville, MD). RCN-9 cells were maintained in DMEM with 10% FCS and 0.2 mmol $l^{-1}$l L-glutamine (Sigma Chemical Co, St Louis, MO, USA). For treatment with Kupffer cell-conditioned media (KCM), RCN-9 cells were cultured in 75-$cm^2$ flasks, and incubated for 48 h in freshly prepared KCM under standard incubator conditions.

### Induction of hepatic metastases

Tumour cell suspensions were prepared by enzymatic detachment of the RCN-9 cells with trypsin-EDTA solution (Sigma Chemical Co, St Louis, MO, USA) at room temperature. After centrifugation, cell concentrations were re-suspended in DMEM to the required concentration. Induction of hepatic metastases was performed by infusing RCN-9 cell suspensions to rat livers (Okuno et al, 1998). Briefly, a laparotomy was performed under ether anaesthesia and a loop of the small intestine was exposed. A suspension of $1 \times 10^6$ RCN-9 cells in 0.5 ml DMEM was slowly injected into a mesenteric vein under microscopic vision with a 0.4 × 12 mm needle, followed by ligation of the vein. The animals were sacrificed after 14 days, and macroscopic metastases, which appeared as pale nodules larger than 5 mm under the liver capsule and often confluent, were searched and counted. Untreated RCN-9 cells were infused to cirrhotic livers of rats induced by $CCl_4$ ($n$ = 20), and to normal livers of healthy control rats ($n$ = 20). Additionally, RCN-9 cells treated with KCM from cirrhotic rat livers were infused to normal livers of healthy rats ($n$ = 20).

### Isolation of tumour-infiltrating lymphocytes

Tumour-infiltrating lymphocytes in metastatic lesions of non-cirrhotic livers were isolated as described previously (Yoong and Adams, 1998). Liver tissues with metastatic tumours were cut into small pieces, and were added to medium without FCS but with collagenase II (0.05%), DNase (0.002%) and hyaluronidase (2.5 U $ml^{-1}$; Sigma Chemical Co, St Louis, MO, USA), which were then incubated at 37˚C 1 h on a shaker. The tissue digest was then passed through a nylon mesh to obtain a single cell suspension that was washed with PBS until the supernatant became clear. The single cell suspension was layered onto Ficoll Hypaque (Lymphoprep, Biovalley Co, Rockville, MD) and centrifuged at 1200 ***g*** for 30 min at room temperature. Lymphocytes and tumour cells were recovered from the interface.

For further isolation of infiltrating lymphocytes, the cells were incubated with biotin-labelled anti-CD5 (Serotec Camon Labor-Service GmbH, Wiesbaden, Germany) for 45 min at 4˚C, washed twice in MACS buffer (0.5% BSA, EDTA and $Mg^{2+}$ and $Ca^{2+}$ free PBS) and incubated with streptavidin-MACS beads (Miltenyi Biotec, Auburn, CA) for 15 min at 4˚C. They were washed twice in MACS buffer and run on a MACS column. The method resulted in an enrichment of T lymphocytes by up to 90% or more.

### In-vitro cytotoxic assay

The in-vitro cytotoxic effect of Kupffer cells or tumour-infiltrating lymphocytes (TILs) on RCN-9 cells was individually assessed by radioactive release assays. Tumour target cells, RCN-9, in their exponential growth phase were labelled by incubation for 24 h in medium containing 0.2 μCi $ml^{-1}$ [$^3$H]-thymidine (>2500 Ci $mmol^{-1}$ Amersham-Buchler, Braunschweig, Germany). After labelling, RCN-9 cells were washed 3 times with HBSS to remove unbound radioisotope, harvested by a brief trypsinization, and re-suspended in DMEM with 10% FCS.

Then target tumour cells were seeded into 96-well flat-bottom-microtiter plates (Nunc, Wiesbaden, Germany) at a density of $1 \times 10^4$ $well^{-1}$ and incubated with medium alone for 24 h at 37˚C under 5% $CO_2$. In some experiments, target cells were pre-incubated in the presence of the antagonistic anti-FasR-blocking antibody clone ZB4 (Biovalley Co, Rockville, MD) at a final concentration of 2 μg $ml^{-1}$ for 1 hour. Freshly isolated TILs or Kupffer cells were added as effector cells at various effector-to-target ratios, and the final volume per well was 200 μl DMEM with 10% FCS. Radiolabelled target cells were also plated alone as a negative control. Effector and target cells (with various E:T ratios) were co-cultured for 72 h for Kupffer cell-mediated cytotoxicity assay and for 24 h for TIL-mediated one. After being washed twice with PBS, the adherent viable cells were lysed with 0.1 ml of 0.1 N KOH, and the macromolecular DNA was transferred and bound onto a glass fibre filter mat (Wallac, Freiburg, Germany). Fragmented DNA was washed through the filter by 4 washes of 0.25 ml water. The radioactivity of intact chromosomal DNA retained on each filter was measured by a liquid scintillation counter (LKB/Wallac, Freiburg, Germany). Cytotoxicity was calculated using the following formula:

$$\text{Cytotoxicity (\%)} = ((S - E) / S) \times 100$$

Where E (experimental) was the cpm of retained DNA in the presence of effector cells, and S (spontaneous) is the one in the absence of effector cells.

 *© 2001 Cancer Research Campaign*

### Assessment of FasL-induced apoptosis

Sensitivity of RCN-9 cells to FasL-induced apoptosis was further determined by incubation with the recombinant human soluble (rhs) Fas ligand (Alexis, Läufelfingen, Switzerland) (Müschen et al, 1998) at concentrations of 10, 100, 500 and 1000 ng $ml^{-1}$, or in DMEM with 10% FCS as control. After 24 h of rhsFasL incubation, the number of apoptotic cells was determined by the terminal deoxynucleotide transferase-mediated dUTP nick end labelling (TUNEL) assay (Boehringer Mannheim GmbH, Mannheim, Germany) and flow cytometry. Briefly, tumour cells were permeabilized with 0.1% Triton X-100 for 5 minutes, and incubated for 60 min at 37˚C in a labelling TUNEL-reaction mixture containing terminal deoxynucleotidyl transferase (TdT) reaction buffer (10.0 µl), bromodeoxyuridine (Br-dUTP; 8.0 µl), $H_2O$ (32.25 µl), and TdT (0.75 µl). The reaction was then terminated with addition of a rinse buffer. Incorporated Br-dUTP was detected after the addition of fluorescein-labelled anti-bromodeoxyuridine antibody (5.0 µl) and incubation for 30 min at room temperature in the dark. Flow cytometric analysis was performed, and data were expressed as percentage of TUNEL-positive tumour cells.

### Immunofluorescence analysis

Quantification of Fas receptors expressed on colon cancer cells was performed by immunofluoresence flow cytometric analysis. After washing twice, RCN-9 cells were incubated with anti-rat FasR mAb (M20, Santa Cruz Biotechnology, Santa Cruz, CA) for 30 min at 4˚C and washed in PBS containing 2% FCS. FITC-conjugated secondary antibody (Dako Corp, Carpinteria, CA) was added to the cells for 30 min at 4˚C. Cells were washed again in PBS containing 2% FCS, and the intensity of fluorescence was analysed with FACScan flow cytometer and LYSIS II software (Nippon Becton Dickinson, Tokyo, Japan). Isotype-matched control antibody was used to determine nonspecific binding. PHA-activated peripheral blood lymphocytes (PBL) isolated from normal rats served as positive controls for FasR expression. A total of 10 000 cells were examined for each determination. Data were expressed as relative fluorescence intensity (RFI = mean fluorescence intensity of cells stained with anti-Fas mAb/mean fluorescence intensity of cells stained with control mAb).

### RT-PCR assays

FasR mRNA expression in colon cancer cells and TNFα as well as IL-1α mRNA expression in Kupffer cells from cirrhotic and normal rat livers were monitored by semi-quantitative RT-PCR. RNA extraction and reverse transcription were performed as previously described (Boekelheide et al, 1998). PCR was performed on cDNA using the primers (sense and anti-sense) for the amplification of FasR, TNFα, IL-1α and a housekeeping gene, *β-actin*. All primers were designed according to the published sequences (Nadeau et al, 1995; Boekelheide et al, 1998).

Thermal cycling was as follows: denaturation at 94˚C for 1 min, annealing at 65˚C for 1 min, and extension at 72˚C for 1 min. 40 cycles were performed for FasR, 35 cycles for TNFα and IL-1α, and 28 cycles for the β-actin PCR. Primers were used at a final concentration of 0.1 µM each, dNTPs at 50 µm, $MgCl_2$ at 1.5 mM, Taq DNA polymerase at 1.0 µ per 50 µl reaction. PCR products were separated by electrophoresis in 2% agarose gels and stained with ethidium bromide, and products of 682 bp were predicted for FasR, 295 bp for TNFα, 623 for IL-1α and 762 bp for β-actin, respectively. The target bands were analysed densitometrically by using a GS-700 Imaging Densitometer (BioRad, Hercules, CA). Cytokine cDNA was semiquantitated by densitometric comparison with β-actin from the same sample. PHA-activated PBL isolated from normal rats served as positive controls for FasR mRNA expression.

### Statistical analysis

Data were given as mean ± SEM ($n \geq 5$). Statistical analysis was performed by one-way analysis of variance (ANOVA) and comparisons among groups were performed by Bonferroni's multiple-comparison *t*-test. Significance level was set at $P < 0.05$.

### RESULTS

### Metastasis formation in cirrhotic and normal rat livers

After 14 days of inoculation with untreated RCN-9 cells, liver metastases were macroscopically visible in 18 out of 20 (90%) rats without liver cirrhosis, while only 8 out of 20 (40%) rats with liver cirrhosis developed hepatic metastases (Table 1, $P < 0.05$). However, when RCN-9 cells pre-treated with KCM from cirrhotic rat livers (cirrhotic KCM) were used to inoculate normal rat livers, the number of rats developing hepatic metastases fell to 11 out of 20 (55%, $P < 0.05$), which was comparable to metastasis formation of untreated cells in cirrhotic livers ($P > 0.05$). Metastases were never observed at the injection site or at any other organ within the time of this study.

### Direct cytotoxicity of Kupffer cells on tumour cells

Co-cultivation of RCN-9 cells with Kupffer cells isolated from both cirrhotic and normal rat livers did not result in significant difference in specific killing (% cytotoxicity) at any E:T ratio ($P > 0.05$, Figure 1). Additionally, despite the increase of E:T ratio, the cytotoxicity of cirrhotic Kupffer cells on RCN-9 cells remained stable at a low level ($P > 0.05$).

### Effect of cirrhotic KCM on TIL-mediated tumour cytotoxicity

As shown by [$^3$H]-release assay (Figure 2A), treatment with normal KCM did not increase the specific killing in RCN-9 cells mediated

**Table 1** A comparison of metastasis formation of cirrhotic KCM treated and untreated RCN-9 colon carcinoma cells in cirrhotic and normal rat livers

| Animal groups | Metastasis (%) | Non-metastasis (%) |
|---|---|---|
| Untreated RCN-9 cells inoculated to normal livers ($n$ = 20) | 18 (90%) | 2 (10%) |
| Untreated RCN-9 cells inoculated to cirrhotic livers ($n$ = 20) | 8 (40%)* | 12 (60%)* |
| Cirrhotic KCM treated RCN-9 cells inoculated to normal livers ($n$ = 20) | 11 (55%)* | 9 (45%)* |

*$P < 0.05$ as compared with the animals with untreated RCN-9 cells inoculated to normal livers.

© 2001 Cancer Research Campaign 

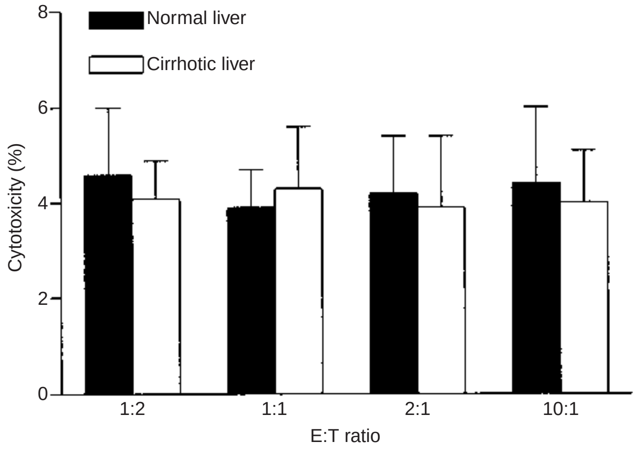

**Figure 1** Kupffer cell-mediated cytotoxicity against RCN-9 cells. [$^3$H]-labelled RCN-9 cells (target cells) were co-cultivated for 72 h with Kupffer cells (effector cells) isolated from normal or cirrhotic rat liver at different E:T ratios. Results were expressed as the mean (± SEM) cytotoxicity (%) of Kupffer cells ($n \geq 5$)

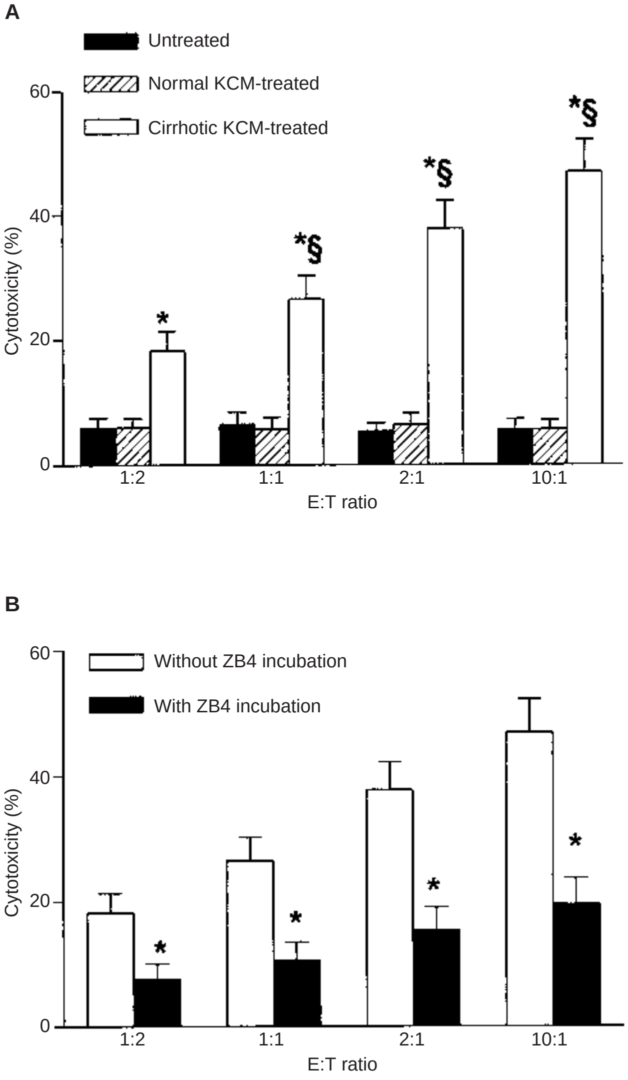

**Figure 2** TIL-mediated cytotoxicity against RCN-9 cells. Results were expressed as the mean (± SEM) cytotoxicity (%) of Kupffer cells ($n \geq 5$). **(A)** Untreated, normal KCM-treated or cirrhotic KCM-treated RCN-9 cells (target cells) labelled with [$^3$H] were co-cultivated for 48 h with tumour-infiltrating lymphocytes (effector cells) at different E:T ratios. $^*P < 0.01$ as compared with the cytotoxicity against untreated RCN-9 cells at the same E:T ratio; $^{§}P < 0.05$ as compared with the cytotoxicity against cirrhotic KCM-treated RCN-9 cells at a lower E:T ratio. **(B)** Cirrhotic KCM-treated RCN-9 cells (target cells) without or with ZB4 pre-incubation were co-cultivated for 48 h with tumour-infiltrating lymphocytes at different E:T ratios. $^*P < 0.01$ as compared with KCM-treated RCN-9 cells without ZB4 pre-incubation at the same E:T ratio by co-culture of TILs as compared with untreated cells ($P > 0.05$), and the cytotoxicity of TILs to both untreated and normal KCM-treated cells remained stable at low levels as the E:T ratio increased ($P > 0.05$). However, treatment with cirrhotic KCM sensitized the tumour cells to TIL- mediated cytotoxicity, as the lytic effect in these cells was significantly more intense than untreated cells at the same E:T ratio ($P < 0.01$), reaching 46.9 ± 5.3% at E:T ratio of 10:1. Furthermore, increases of E:T ratio resulted in elevation of TIL-mediated cytotoxicity in a dose-dependent manner ($P < 0.05$).

To determine whether TIL-mediated cell lysis was FasR-mediated, we pre-incubated target tumour cells with 2 μg ml$^{-1}$ of the antagonistic anti-FasR blocking antibody clone ZB4, which remained in the culture medium during exposure to isolated lymphocytes. ZB4 treatment specifically inhibited TIL-mediated cytotoxicity to cirrhotic KCM-treated RCN-9 cells ($P < 0.01$) (Figure 2B). However such treatment did not modify the cytolytic activity of lymphocytes toward normal KCM-treated cells or untreated cells (data not shown). Furthermore, as reported elsewhere the ZB4 monoclonal antibody alone did not influence cytolysis of RCN-9 cells (not shown).

### Effect of cirrhotic KCM on FasL-induced apoptosis

After incubation for 24 h in the presence of rhsFasL at concentrations up to 1 μg ml$^{-1}$, a dose-dependent increase of apoptotic cells was observed in tumour cells treated with cirrhotic KCM (Figure 3). Percentage of cell death was elevated to 12.6 ± 1.6 at 10 ng ml$^{-1}$ of rhsFasL, which was significantly higher than control ($P < 0.01$), and reached 55.4 ± 7.1 at 1 μg ml$^{-1}$ of rhsFasL. In contrast, apoptotic percentage of RCN-9 cells treated with normal KCM, as well as untreated cells, remained stable with the increase of rhsFasL concentration. Even at a concentration (1 μg ml$^{-1}$) 100 times higher than the one necessary to induce apoptosis in rat hepatocytes (Boekelheide et al, 1998), percentage of apoptotic cancer cells in this group did not differ from control ($P > 0.05$).

### Effect of cirrhotic KCM on Fas receptor expression of tumour cells

In order to evaluate whether the sensitizing effect of cirrhotic KCM on colon cancer cells was mediated by up-regulating Fas receptor levels on tumour cell surface, we detected Fas receptor expression on RCN-9 cells at both protein and mRNA levels. As shown by immunofluoresence flow cytometry analysis (Figure 4), RCN-9 cells treated with normal KCM, as well as untreated cells, failed to express significant amount of Fas receptors (RFI 3.13 ± 1.74 for normal KCM-treated cells; 4.27 ± 2.05 for untreated cells), which was quantitatively much lower than that seen in PHA-activated PBLs serving as a positive control (53.5 ± 16.4; $P < 0.01$). In contrast, RCN-9 cells showed a prominent increase in FasR surface density after exposure to cirrhotic KCM, with an RFI of 127.7 ± 24.6 which was higher than that of the positive control ($P < 0.05$). This paralleled the changes of FasR mRNA expression in RCN-9 cells upon treatment with cirrhotic KCM, as

© 2001 Cancer Research Campaign

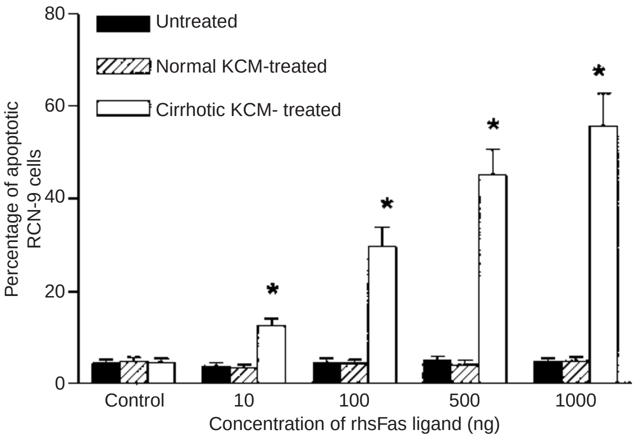

**Figure 3** Apoptosis of untreated, normal KCM-treated or cirrhotic KCM-treated RCN-9 cells induced by treatment with different concentrations of rhsFasL. Results were expressed as mean percentage of apoptotic RCN-9 cells (± SEM) ($n \geq 5$). RCN-9 cells exposed to media alone served as control. *$P < 0.01$ as compared with the apoptotic percentage of RCN-9 cells exposed to a lower concentration of rhsFasL

these cells expressed much higher levels of FasR mRNA (3.26 ± 1.49 times β-actin) in comparison to cells treated with normal KCM (0.54 ± 0.27 times β-actin) and untreated cells (0.39 ± 0.15 times β-actin) ($P < 0.01$).

### Cytokine expression in cirrhotic Kupffer cells

To further determine whether activated Kupffer cells isolated from cirrhotic rat livers express proinflammatory factors, we determined the mRNA expression of TNFα and IL-1α in these cells. In Kupffer cells isolated from normal rat livers, only basal expression of TNFα (0.28 ± 0.06 times β- actin) and IL-1α (0.42 ± 0.14 times β-actin) mRNA was observed. In sharp contrast, activated Kupffer cells isolated from cirrhotic rat livers expressed much higher mRNA levels of TNFα (2.76 ± 0.53 times β-actin; $P < 0.01$) and IL-1α (3.07 ± 0.84 times β-actin; $P < 0.01$) respectively as compared to normal ones.

### DISCUSSION

In the present study, we found that colon cancer cells preferentially metastasized to normal rat livers, and less frequently to cirrhotic ones induced by $CCl_4$. Our results support previous clinical data, and confirm that cirrhotic livers are not a favourable environment for metastatic tumour cells (Seymour and Charnley, 1999). More interestingly, treatment of colon cancer cells with Kupffer cell-conditioned media from cirrhotic livers dramatically reduced the occurrence of metastases in normal rat livers to the extent that was comparable to metastases in cirrhotic livers. This phenomenon suggests that Kupffer cells from cirrhotic livers contribute to the inhibition of hepatic colonization of colon cancer cells.

It has been shown that during the development of the inflammatory process in cirrhotic livers, Kupffer cells proliferate extensively and are activated to produce proinflammatory cytokines, which play a central role in mediating cytotoxicity to parenchymal cells and inducing the inflammatory response (Gressner, 1996). Hence, it is reasonable to extrapolate that Kupffer cells from cirrhotic livers directly kill colon cancer cells, and thus impede the formation of liver metastases. However, Kupffer cells from cirrhotic livers were not effective in killing colon cancer cells directly in our co-culture system even at a high effector to target ratio (40:1). Similarly, Hauptmann et al (1993) have also demonstrated that activated macrophages are not effective in inhibiting the proliferation of tumour cells in vitro. These findings suggest that the hindering effect of Kupffer cells from cirrhotic livers on hepatic colonization of colon cancer cells does not depend on direct tumour cytotoxicity.

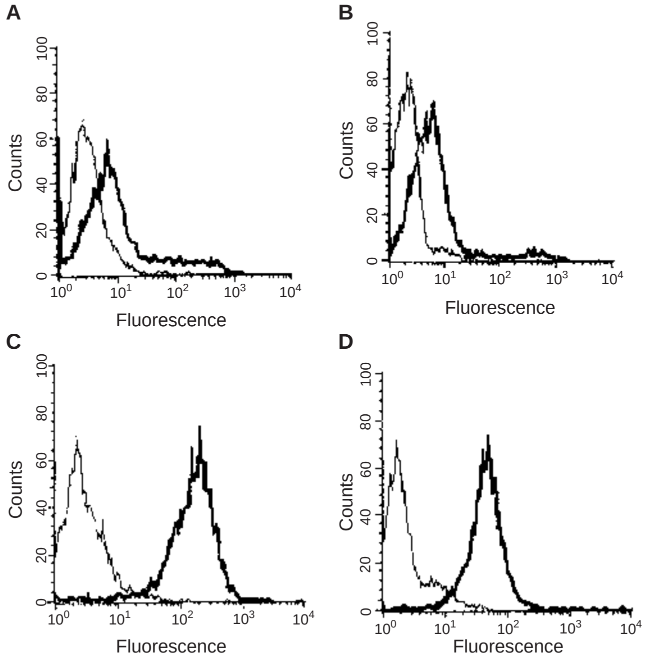

**Figure 4** Flow cytometric analysis of FasR expression on untreated (**A**), normal KCM-treated (**B**) and cirrhotic KCM-treated (**C**) RCN-$H_4$ cells, as well as PHA-activated PBL (positive control) (**D**) using anti-FasR mAb (bold peaks) or an irrelevant antibody (isotype control) for the determination of background staining (thin-line peaks)

Nevertheless, our study showed that treatment of colon cancer cells with cirrhotic KCM, but not with normal KCM, resulted in an increase of cytotoxicity mediated by tumour-infiltrating lymphocytes in a dose-dependent manner. This suggests that despite inefficacy in direct tumour cell killing, Kupffer cells from cirrhotic livers are able to sensitise colon cancer cells to TIL-mediated lysis.

It has been well established that FasR and FasL interaction is a major mechanism of T-cell-mediated cytotoxicity (Nagata, 1997). Tumour-infiltrating lymphocytes (TILs) of colon cancers, as well as metastatic ones, express Fas ligand on their cell surfaces (O'Connell et al, 1998; Yoong et al, 1999). In order to confirm that the present TIL-induced cytotoxicity towards cirrhotic KCM-treated colon cancer cells was FasR-mediated, an antagonistic anti-FasR–blocking antibody which alone does not modify cytolysis (Houghton et al, 1997), was used before the cytotoxic assay. Application of the antibody completely abolished TIL-mediated cytotoxicity to target tumour cells, suggesting that this kind of cell killing involves Fas–FasL interaction. In addition, the sensitivity of cirrhotic KCM-treated colon cancer cells to FasR-mediated apoptosis was further corroborated by increased apoptosis induced by recombinant human soluble Fas ligand in a dose-dependent manner.

© 2001 Cancer Research Campaign 

In contrast, we found that normal KCM-treated or untreated RCN-9 cells are refractory to FasR-mediated apoptosis, which is in agreement with findings in human colon cancer cells (Möller et al, 1994; O'Connell et al, 1996; Krammer et al, 1998; O'Connell et al, 1998). FasR-resistance of colon cancer cells is thought to be involved in the mechanism of immune evasion, and associated with their metastatic phenotype (Möller et al, 1994; O'Connell et al, 1998; Yoong et al, 1999). Restoration of colon cancer cell sensitivity to Fas-mediated apoptosis is able to facilitate them to TIL-mediated killing, and has been proposed to be an effective immunotherapy for containing the malignancy (Tamura et al, 1995; Houghton et al, 1997; Micheau et al, 1997; Bonnotte et al, 1998; Fukazawa et al, 1999). Thus, sensitization of colon cancer cells to Fas-mediated apoptosis by cirrhotic Kupffer cells, as demonstrated in the present study presumably contributes to the immunologic impedance of cirrhotic livers against metastasis formation of colorectal cancers.

As a sufficient level of Fas receptors has been shown to be required for the induction of apoptosis in colonic epithelial cells (Tillman et al, 1998; von Reyher et al, 1998), we further studied the mechanism for FasR-sensitivity alternation in differently treated colon cancer cells by examining the levels of their FasR expression. We found that the untreated RCN-9 cells, as well as the ones treated with normal KCM, expressed Fas receptor mRNA and protein at much lower levels than positive controls. Similarly, Möller et al (1994), have demonstrated that expression of Fas protein in the colon is progressively reduced during the transformation of normal epithelium to benign neoplasms, adenocarcinomas, and ultimately, to metastases, as only 10% of adenomas exhibited reduced Fas expression compared with 88% of carcinomas, and complete absence of Fas was most common in metastatic tumours. Hence, loss of Fas expression contributes to the unresponsiveness of colon cancer cells to FasR-mediated apoptosis. Nevertheless, our findings showed that treatment with cirrhotic, instead of normal KCM tremendously up-regulated FasR surface density and FasR mRNA expression in colon cancer cells to the level similar to positive control, which paralleled its sensitizing effect on FasR-mediated apoptosis. Houghton et al (1997) have suggested that signal transduction of FasR-mediated apoptosis in colon cancer cells is intact and functional, and restoration of sufficiently high levels of Fas receptors allows transduction of the signal from the cell surface to induce apoptosis. This position is supported by the findings that a variety of chemotherapeutic drugs (Micheau et al, 1997, 1999) and anti-tumour cytokines (Möller et al, 1994), such as TNF-α and IFN-γ, exhibit an indirect cytotoxic effect on colon cancer cells by enhancing their expression of Fas receptors, and thus preparing the tumour cells to be eliminated by immune system using a Fas-dependent pathway. Thus, the up-regulation of Fas receptor expression on colon cancer cells upon treatment with cirrhotic KCM revealed in the current study probably contributes to sensitization of the tumour cells to FasR-mediated apoptosis induced by Kupffer cells from cirrhotic livers.

It has been known that Kupffer cells in cirrhotic livers function differently from the macrophages in other tissue or in non-cirrhotic livers by releasing pro-inflammatory cytokines (Gressner, 1996). Our present study confirmed that Kupffer cells isolated from cirrhotic rat livers expressed elevated levels of TNFα and IL-1α mRNA. As tumour necrosis factor and interleukin-1 have been shown to enhance the level of FasR expression on cell surfaces (Litton et al, 1996; Moulian et al, 1999), we suggest that over-production of these cytokines by Kupffer cells in cirrhotic livers probably accounts for their modulating effects on colon cancer cells. However, further studies are needed to assess the regulating effects of cytokines and growth factors produced by Kupffer cells in cirrhotic liver on FasR-sensitivity of colon cancer cells.

In summary, our study presents the first experimental evidence that alterations in liver-associated immunity during hepatic cirrhosis provides an immunologic impediment to hepatic colonization of colon cancers. We concluded that Kupffer cells in cirrhotic livers sensitize metastatic colon cancer cells to FasR-mediated apoptosis by up-regulating their expression of Fas receptors, which thus prepare the malignancies to be eliminated by tumour-infiltrating lymphocytes. Hence, activation of Kupffer cells during hepatic cirrhosis on one hand results in tissue damage and fibrogenesis in livers, but on the other hand inhibits the hepatic matastasis formation of colon cancers.

### REFERENCES

Armendáriz-Borunda J, Greenwel P and Rojkind M (1989) Kupffer cells from $CCl_4$-treated rat livers induce skin fibroblast and liver fat-storing cell proliferation in culture. *Matrix* **9**: 150–158

Boekelheide K, Lee J, Shipp EB, Richburg JH and Li G (1998) Expression of Fas system-related genes in the testis during development and after toxicant exposure. *Toxicol Lett* **102–103**: 503–508

Bonnotte B, Favre N, Reveneau S, Micheau O, Droin N, Garrido C, Fontana A, Chauffert B, Solary E and Martin F (1998) Cancer cell sensitization to Fas-mediated apoptosis by sodium butyrate. *Cell Death Differ* **5**: 480–487

Fukazawa T, Fujiwara T, Morimoto Y, Shao J, Nishizaki M, Kadowak S, Hizuta A, Owen-Schaub LB, Roth JA and Tanaka N (1999) Differential involvement of the CD95 (Fas/APO-1) receptor/ligand system on apoptosis induced by the wild-type p53 gene transferred human cancer cells. *Oncogene* **18**: 2189–2199

Gressner AM (1996) Mediators of hepatic fibrogenesis. *Hepatogastroenterol* **43**: 92–103

Griffini P, Smorenburg SM, Vogels IMC, Tigchelaar W and van Noorden, CJF (1996) Kupffer cells and pit cells are not effective in the defense against experimentally induced colon carcinoma metastasis in rat liver. *Clin Exp Metast* **14**: 367–380

Hauptmann S, Zwadlo KG, Jansen M, Klosterhalfen B and Kirkpatrick CJ (1993) Macrophages and multicellular tumor spheroids in co-culture: a three-dimensional model to study tumor-host interactions. Evidence for macrophage-mediated tumor cell proliferation and migration. *Am J Pathol* **143**: 1406–1415

Hayashi S, Masuda H and Shigematsu M (1997) Liver metastasis rate in colorectal cancer patients with fatty liver. *Hepatogastroenterol* **44**: 1069–1075

Houghton JA, Harwood FG, Gibson AA and Tillman DM (1997a) The Fas signaling pathway is functional in colon carcinoma cells and induces apoptosis. *Clin Cancer Res* **3**: 2205–2209

Houghton JA, Harwood FG and Tillman DM (1997b) Thymineless death in colon carcinoma cells is mediated via Fas signaling. *Proc Natl Acad Sci USA* **94**: 8144–8149

Inoue Y, Kashima Y, Aizawa K and Hatakeyama K (1991) A new rat colon cancer cell line metastasizes spontaneously: Biologic characteristics and chemotherapeutic response. *Jpn J Cancer Res* **82**: 90–98

Krammer PH, Galle PR, Möller P and Debatin KM (1998) CD95 (APO-1/Fas)-mediated apoptosis in normal and malignant liver, colon, and hematopoietic cells. *Adv Cancer Res* **75**: 251–273

Litton MJ, Dohlsten M, Lando PA, Kalland T, Ohlsson L, Andersson J and Andersson U (1996) Antibody-targeted superantigen therapy induces tumor-infiltrating lymphocytes, excessive cytokine production, and apoptosis in human colon carcinoma. *Eur J Immunol* **26**: 1–9

Micheau O, Solary E, Hammann A, Martin F and Dimanche-Boitrel MT (1997) Sensitization of cancer cells treated with cytotoxic drugs to Fas-mediated cytotoxicity. *J Natl Cancer Inst* **89**: 783–789

Micheau O, Hammann A, Solary E and Dimanche-Boitrel MT (1999) STAT-1-independent upregulation of FADD and procaspase-3 and -8 in cancer cells treated with cytotoxic drugs. *Biochem Biophys Res Commun* **256**: 603–607

*© 2001 Cancer Research Campaign*

Möller P, Koretz K, Leithauser F, Bruderlein S, Henne C, Quentmeier A and Krammer PH (1994) Expression of APO-1 (CD95), a member of the NGF/TGF receptor superfamily, in normal and neoplastic colon epithelium. *Int J Cancer* **57**: 371–377

Moulian N, Renvoizé C, Desodt C, Serraf A and Berrih-Aknin S (1999) Fuctional Fas expression inhuman thymic epithelial cells. *Blood* **93**: 2660–2670

Müschen M, Warskulat U, Douillard P, Gilbert E and Häussinger D (1998) Regulation of CD95 (APO-1/Fas) receptor and ligand expression by lipopolysaccharide and dexamethasone in parenchymal and nonparenchymal rat liver cells. *Hepatology* **27**: 200–208

Nadeau KC, Azuma H and Tilney NL 1995 Sequential cytokine dynamics in chronic rejection of rat renal allografts: Roles for cytokines RANTES and MCP-1. *Proc Natl Acad Sci USA* **92**: 8729–8733

Nagata S (1997) Apoptosis by death factor. *Cell* **88**: 355–65

O'Connell J, O'Sullivan GC, Collins JK and Shanahan F (1996) The Fas counterattack: Fas-mediated T cell killing by colon cancer cells expressing Fas ligand. *J Exp Med* **184**: 1075–1082

O'Connell J, Bennett MW, O'Sullivan GC, Roche D, Kelly J, Collins JK and Shanahan F (1998) Fas ligand expression in primary colon adenocarcinomas: evidence that the Fas counterattack is a prevalent mechanism of immune evasion in human colon cancer. *J Pathol* **186**: 240–246

Okuno K, Hirai N, Lee YS, Kawai I, Shigeoka H and Yasutomi M (1998) Involvement of liver-associated immunity in hepatic metastasis formation. *J Surg Res* **75**: 148–152

Seymour K and Charnley RM (1999) Evidence that metastasis is less common in cirrhotic than normal liver: a systematic review of post-mortem case-control studies. *Br J Surg* **86**: 1237–1242

Tamura T, Aoyama N, Saya H, Haga H, Futami S, Miyamoto M, Koh T, Ariyasu T, Tachi M, Kasuga M and Takahashi R (1995) Induction of Fas-mediated apoptosis in p53-transfected human colon carrcinoma cells. *Oncogene* **11**: 1939–1946

Thomas C, Nijenhuis AM, Timens W, Kuppen PJK, Daemen T and Scherphof GL (1993) Liver metastasis model of colon cancer in the rat: immunohistochemical characterization. *Invas Metast* **13**: 102–112

Tillman DM, Harwood FG, Gibson AA and Houghton JA (1998) Expression of genes that regulate Fas signalling and Fas-mediated apoptosis in colon carcinoma cells. *Cell Death Differ* **5**: 450–457

Utsunomiya T, Saitsu H, Saku M, Yoshida K, Matsumata T, Shimada M and Sugimachi K (1999) Rare occurrence of colorectal cancer metastasis in livers infected with Hepatitis B or C virus. *Am J Surg* **177**: 279–281

Von Reyher U, Strater J, Kittstein W, Gschwendt M, Krammer PH and Möller P (1998) Colon carcinoma cells use different mechanisms to escape CD95-mediated apoptosis. *Cancer Res* **58**: 526–534

Yoong KF and Adams DH (1998) Interleukin-2 restores CD3 zeta chain expression but fails to generate specific cytolytic activity in tumor infiltrating lymphocytes isolated from patients with hepatic colorectal metastases. *Br J Cancer* **77**: 1072–1081

Yoong KF, Afford SC, Randhawa S, Hubscher SG and Adams DH (1999) Fas/Fas ligand interaction in human colorectal hepatic metastases. *Am J Pathol* **154**: 693–703

*© 2001 Cancer Research Campaign*
